# Associations Between Initial Glycated Hemoglobin at Diabetes Diagnosis, Longitudinal Changes and Medication Effects, and the Severity and Progression of Sight‐Threatening Diabetic Retinopathy

**DOI:** 10.1002/kjm2.70148

**Published:** 2025-12-17

**Authors:** Yu‐Chi Sung, Yu‐Kuang Chen, Ching‐An Chen, Pei‐Kang Liu, Kai‐Chun Cheng, Wei‐Shan Chang, Chia‐Li Tseng, Hui‐Min Hsieh, Shwu‐Jiuan Sheu

**Affiliations:** ^1^ Department of Ophthalmology Kaohsiung Medical University Hospital Kaohsiung Taiwan; ^2^ Department of Ophthalmology, School of Medicine College of Medicine, Kaohsiung Medical University Kaohsiung Taiwan; ^3^ Division of Medical Statistics and Bioinformatics, Department of Medical Research Kaohsiung Medical University Hospital, Kaohsiung Medical University Kaohsiung Taiwan; ^4^ Department of Public Health Kaohsiung Medical University Kaohsiung Taiwan; ^5^ Center for Big Data Research Kaohsiung Medical University Kaohsiung Taiwan

**Keywords:** diabetic retinopathy, glycated hemoglobin (HbA1c), sight‐threatening diabetic retinopathy (STDR), type 2 diabetes mellitus (T2DM)

## Abstract

Diabetic retinopathy (DR) is a leading cause of vision impairment in patients diagnosed with Type 2 diabetes mellitus (T2DM). Awareness of risk factors is necessary to reduce or prevent harmful effects of DR such as irreversible vision loss, and to help ensure early treatment. This study aimed to evaluate associations between initial HbA1c at diabetes diagnosis, longitudinal changes, and medication adherence (MR), and the severity and progression of sight‐threatening diabetic retinopathy (STDR). From July 2022 to January 2024, 300 patients with T2DM aged ≥ 20 years were recruited, and the data of 178 patients with complete data were analyzed, focusing on initial HbA1c levels at DM diagnosis, subsequent HbA1c changes at ophthalmologic visits, and MR using the Taiwanese version of the Morisky Medication Adherence Scale‐8 (MMAS‐8). Patients with persistently high or fluctuating HbA1c levels had a higher risk of severe DR compared to those with consistently low HbA1c. High initial HbA1c was more strongly associated with DR severity in females, patients aged < 65 years, and those without diet or exercise control. Poor or moderate MR was associated with higher HbA1c at follow‐up and increased STDR risk. In contrast, patients aged ≥ 65 years were less likely to develop severe DR. Findings of this study accentuate the importance of initial glycemic control and HbA1c trends during ophthalmologic care in managing DR progression, suggesting that patients with high initial HbA1c may benefit from closer early ophthalmic monitoring.

## 1 Introduction

Diabetic retinopathy (DR) is a leading cause of vision impairment in individuals with diabetes mellitus (DM). By 2030, over 25% of the global diabetic population is estimated to be at risk of vision loss from DR, resulting in a significant burden on healthcare systems [1]. Awareness of risk factors is necessary to reduce or prevent harmful effects of DR such as irreversible vision loss and to help ensure early treatment.

Previously reported major risk factors for DR progression include diabetes duration and high HbA1c levels. Other contributing factors are uncontrolled fasting blood sugar, hypertension, hyperlipidemia, pregnancy, nephropathy, obesity, and genetic predisposition [2, 3, 4, 5, 6, 7]. In addition to early detection and treatment, strict glycemic control is crucial for preventing DR‐related vision loss. The UK Prospective Diabetes Study (UKPDS) found that a 1% reduction in mean HbA1c is associated with a 37% decrease in the risk of DM‐related microvascular complications [3]. Other reports show that non‐compliance with diabetes medications may worsen comorbidities such as diabetic neuropathy, nephropathy, cardiovascular disease, and increase mortality [8, 9, 10]. Advanced treatments such as photocoagulation, intravitreal injections, and vitrectomy are used for sight‐threatening DR [11]. Since sight‐threatening diabetic retinopathy (STDR) primarily affects working individuals, causing significant social impact nationally [12], STDR risk factors need further investigation [13].

Therefore, this study aimed to evaluate associations between initial HbA1c at diabetes diagnosis, longitudinal changes, medication adherence (MR), and the severity and progression of STDR in patients with Type 2 diabetes mellitus (T2DM), focusing on the predictive value of initial HbA1c at diagnosis and longitudinal changes during ophthalmologic follow‐up.

## 2 Methods

### 2.1 Study Design and Sample

This prospective cohort study recruited 300 patients diagnosed with T2DM from the outpatient department of Kaohsiung Medical University Hospital between July 2022 and January 2024. Patients aged 20 years or older, with a diabetes diagnosis (ICD‐9‐CM codes 250.xx), were included. Those missing HbA1c or blood glucose data at DM diagnosis or the first ophthalmologic visit, which were primarily due to incomplete laboratory testing in earlier medical records or referrals from outside facilities without available biochemical results, rather than differences in disease severity, were excluded. After exclusions, the data of 178 patients were included in the final analysis. Data sources included electronic medical records and surveys.

### 2.2 Main Measures

The primary endpoint was the DR score, reflecting both DR progression and severity. DR severity was graded independently by two experienced ophthalmologists, who were blinded to patients' clinical characteristics and group assignments to minimize bias. Although formal inter‐observer reliability was not calculated, both ophthalmologists adhered to standardized grading protocols to ensure consistency. Each subject's DR score was determined based on the most recent ophthalmologic examination of the worse eye. The DR score was categorized into four levels: (1) Score 0: patients without DR; (2) Score 1: patients with non‐STDR; (3) Score 2: patients with DR who had received ophthalmic intervention, including laser treatment or intravitreal pharmacotherapy; and (4) Score 3: patients with severe DR complications, such as neovascular glaucoma (NVG), or those who underwent vitrectomy for vitreous hemorrhage (VH) or tractional retinal detachment (TRD).

Two key independent variables were used as proxies for the potential effects of diabetes control. The first measure, an objective assessment, evaluated longitudinal HbA1c changes using a cut‐off point of 8%. Changes were assessed from diabetes diagnosis to the first ophthalmologic visit and categorized into four groups: sustained low (maintained at < 8%), improved (decreased from ≥ 8% to < 8%), deteriorated (increased from < 8% to ≥ 8%), and sustained high (maintained at ≥ 8%). The second one, a subjective measure, assessed self‐reported MR using the Taiwanese version of the Morisky Medication Adherence Scale‐8 (MMAS‐8),^1^ as validated previously [14, 15]. The MMAS‐8 questionnaire is used to assess MR behaviors. Sample questions include: “Do you sometimes forget to take your medicine?” and “In the past two weeks, have you failed to take your medicine on time for reasons other than forgetting?” To mitigate “yes‐saying” bias, the questions were carefully designed to avoid socially desirable answers. Adherence was categorized into high adherence and low to moderate adherence based on the validated scoring system. [Correction added on 23 January 2026, following initial online publication: An endnote has been incorporated, and the final two sentences have been revised in this version.]

Baseline demographic and clinical characteristics included sex, age, body mass index (BMI), education level, marital status, diabetes duration, control methods, and chronic comorbid conditions (hypertension, heart disease, stroke, kidney, liver, and endocrine diseases). Patients were categorized into two age groups: < 65 and ≥ 65 years on the basis of commonly used thresholds in epidemiologic and clinical studies of T2DM and DR, which often distinguish between “younger” and “older” adults to account for differences in metabolic profiles, comorbidity burden, and risk of DR progression [16, 17]. Comorbidity scores were calculated as the sum of chronic conditions, excluding respiratory and non‐chronic diseases. Additional variables included the timing of diabetes diagnosis, HbA1c or blood glucose at diagnosis, and data from the first ophthalmologic visit, including management strategies such as exercise and dietary modifications.

### 2.3 Statistical Analysis

Descriptive data are presented as means and standard deviations (SD) for continuous variables and frequencies for categorical variables. Chi‐square tests assessed differences between categorical variables, and independent samples t‐tests compared continuous variables. Univariable and multivariable logistic regression models were used to examine factors associated with DR severity, adjusting for confounders. Crude and adjusted odds ratios (AORs) and 95% confidence intervals (CIs) were presented. A two‐tailed *p*‐value < 0.05 was established as statistical significance. All statistical analyses were performed using SAS version 9.4 (SAS Analytics, Cary, NC, USA).

## 3 Results

### 3.1 Patients' Demographic and Clinical Characteristics

Of 178 patients (50% male, 50% female), 85 (47.75%) were classified as having high MR, while 93 (52.25%) were categorized as having moderate to low adherence. Sex distribution was similar across the adherence groups. No significant differences in gender distribution were observed between the high adherence group (52.94% male, 47.06% female) and the moderate‐to‐low adherence group (47.31% male, 52.69% female) in terms of gender distribution (*p* = 0.453). No significant differences were found in age, BMI, education level, and marital status between the two groups.

Also, no significant between‐group differences were found in DM duration and chronic disease comorbidity scores. Patients with self‐reported exercise habits had significantly higher MR (*p* = 0.017), but no significant differences were noted in diet control and antidiabetic medications between the two groups. High HbA1c (≥ 8%) at DM diagnosis or during ophthalmologic visits tended to have significantly lower MR (Appendix 1).

### 3.2 Follow‐Up Duration

Based on the duration of ophthalmology outpatient (OPH‐OPD) follow‐up, we divided the 178 subjects into two groups: (1) follow‐up less than 5 years and (2) follow‐up more than 5 years. The cut point was determined based on our clinical experience that the most aggressive treatment was done within 5 years, as well as on clinical and epidemiological data showing that the majority of incident STDR cases develop within 5 years after T2DM diagnosis [18, 19]. A total of 119 subjects had less than 5 years of follow‐up, with a minimum duration of 0.5 months and a mean duration of 1.68 ± 1.17 years, while 59 subjects had more than 5 years of follow‐up, with a mean duration of 8.93 ± 2.87 years. Statistically significant differences were found between the two groups (*p* < 0.001). Mean age in the shorter follow‐up group was 60.01 years and 43.70% were female. Mean diabetes duration was 9.58 years, and the average chronic disease comorbidity score was 1.46 points. In contrast, patients with more than 5 years OPH‐OPD were generally older, with a mean age of 65.03 years and a higher proportion of females (62.71%). Mean diabetes duration in that group was 14.49 years and mean chronic disease comorbidity score was slightly higher at 1.58 points. Younger patients (< 65 years) had significantly higher DR scores regardless of follow‐up time (*p* = 0.015; *p* = 0.009). Blood glucose levels at DM diagnosis correlated significantly and positively with DR scores in both groups regardless of follow‐up time (*p* < 0.001; *p* = 0.002) (Table 1).

**TABLE 1:** Analysis of the association between follow‐up duration and severity of diabetic retinopathy.

	OPH‐OPD f/u duration < 5 years				OPH‐OPD f/u duration ≥ 5 years			
	Overall	DR score 0–1	DR score 2–3	*p*	Overall	DR score 0–1	DR score 2–3	*p*
*N*	119	52 (43.70%)	67 (56.30%)		59	21 (35.59%)	38 (64.41%)	
Sex (*N*, %)				0.396				0.223
Male	67 (56.30%)	27 (51.92%)	40 (59.70%)		22 (37.29%)	10 (47.62%)	12 (31.58%)	
Female	52 (43.70%)	25 (48.08%)	27 (40.30%)		37 (62.71%)	11 (52.38%)	26 (68.42%)	
Mean age (mean ± SD)	60.01 ± 11.39	62.77 ± 9.04	57.87 ± 12.58	0.015	65.03 ± 9.02	69.10 ± 8.30	62.79 ± 8.71	0.009
Age categories (*N*, %)				0.254				0.060
< 65	71 (59.66%)	28 (53.85%)	43 (64.18%)		30 (50.85%)	7 (33.33%)	23 (60.53%)	
≥ 65	48 (40.34%)	24 (46.15%)	24 (35.82%)		29 (49.15%)	14 (66.67%)	15 (39.47%)	
BMI (mean ± SD)	26.19 ± 4.70	26.16 ± 4.31	26.21 ± 5.01	0.953	26.04 ± 5.43	24.77 ± 2.14	26.74 ± 6.50	0.184
Education level (*N*, %)				0.991				0.884
Illiterate/elementary school	19 (15.97%)	8 (15.38%)	11 (16.42%)		19 (32.20%)	6 (28.57%)	13 (34.21%)	
Junior high school	17 (14.29%)	7 (13.46%)	10 (14.93%)		9 (15.25%)	4 (19.05%)	5 (13.16%)	
High school/technical school	60 (50.42%)	27 (51.92%)	33 (49.25%)		24 (40.68%)	8 (38.10%)	16 (42.11%)	
College or above	23 (19.33%)	10 (19.23%)	13 (19.40%)		7 (11.86%)	3 (14.29%)	4 (10.53%)	
Marital status (*N*, %)				0.557				0.386
Single	18 (15.13%)	6 (11.54%)	12 (17.91%)		6 (10.17%)	2 (9.52%)	4 (10.53%)	
Married/cohabiting	79 (66.39%)	37 (71.15%)	42 (62.69%)		42 (71.19%)	17 (80.95%)	25 (65.79%)	
Divorced/widowed	22 (18.49%)	9 (17.31%)	13 (19.40%)		11 (18.64%)	2 (9.52%)	9 (23.68%)	
Diabetes duration (in years, mean ± SD)	9.58 ± 7.10	9.29 ± 7.31	9.80 ± 6.97	0.698	14.49 ± 8.30	12.95 ± 6.64	15.34 ± 9.06	0.294
Diabetes control methods (*N*, %)								
Exercise	60 (50.42%)	30 (57.69%)	30 (44.78%)	0.162	28 (47.46%)	10 (47.62%)	18 (47.37%)	0.985
Diet	91 (76.47%)	36 (69.23%)	55 (82.09%)	0.101	47 (79.66%)	17 (80.95%)	30 (78.95%)	1.000
Oral hypoglycemic drugs/insulin	118 (99.16%)	52 (100.00%)	66 (98.51%)	1.000	59 (100.00%)	21 (100.00%)	38 (100.00%)	—
Mean chronic disease comorbidity score (mean ± SD)	1.46 ± 0.90	1.48 ± 0.90	1.45 ± 0.91	0.844	1.58 ± 0.81	1.62 ± 0.86	1.55 ± 0.80	0.767
Chronic disease comorbidity score group (*N*, %)				0.891				0.888
0 points	18 (15.13%)	7 (13.46%)	11 (16.42%)		4 (6.78%)	1 (4.76%)	3 (7.89%)	
1–2 points	86 (72.27%)	38 (73.08%)	48 (71.64%)		49 (83.05%)	18 (85.71%)	31 (81.58%)	
3 points or above	15 (12.61%)	7 (13.46%)	8 (11.94%)		6 (10.17%)	2 (9.52%)	4 (10.53%)	
History of chronic diseases (*N*, %)								
Hypertension	83 (69.75%)	32 (61.54%)	51 (76.12%)	0.086	48 (81.36%)	18 (85.71%)	30 (78.95%)	0.730
Heart disease	23 (19.33%)	17 (32.69%)	6 (8.96%)	0.002	13 (22.03%)	6 (28.57%)	7 (18.42%)	0.513
Stroke	8 (6.72%)	4 (7.69%)	4 (5.97%)	0.728	5 (8.47%)	2 (9.52%)	3 (7.89%)	1.000
Kidney‐related diseases	37 (31.09%)	9 (17.31%)	28 (41.79%)	0.005	18 (30.51%)	3 (14.29%)	15 (39.47%)	0.075
Liver‐related diseases	13 (10.92%)	8 (15.38%)	5 (7.46%)	0.237	7 (11.86%)	3 (14.29%)	4 (10.53%)	0.691
Endocrine‐related diseases	10 (8.40%)	7 (13.46%)	3 (4.48%)	0.102	2 (3.39%)	2 (9.52%)	0 (0.00%)	0.123
Others	104 (87.39%)	47 (90.38%)	57 (85.07%)	0.387	54 (91.53%)	20 (95.24%)	34 (89.47%)	0.646
Blood glucose level when DM diagnosed (*N*, %)				< 0.001				0.002
100–200	41 (34.45%)	29 (55.77%)	12 (17.91%)		22 (37.29%)	14 (66.67%)	8 (21.05%)	
200–300	49 (41.18%)	17 (32.69%)	32 (47.76%)		22 (37.29%)	4 (19.05%)	18 (47.37%)	
≥ 300	29 (24.37%)	6 (11.54%)	23 (34.33%)		15 (25.42%)	3 (14.29%)	12 (31.58%)	

Abbreviations: BMI, body mass index; DM, diabetes mellitus.

Among patients with less than 5 years' follow‐up, 63.03% had HbA1c levels below 8% at their first ophthalmology visit, with a higher proportion in the high MR group (53.33%) than in the moderate/low adherence group (46.67%). Patients with low‐to‐moderate MR had significantly higher HbA1c levels (≥ 8%) than those with high MR (*p* = 0.042). A similar trend was seen in the > 5 years' follow‐up group but was not significant. Younger patients (< 65 years) had significantly higher HbA1c levels at ophthalmology visits in both groups. BMI correlated significantly with HbA1c only in the > 5 years group. No significant differences were found in education level, marital status, and diabetes duration between HbA1c levels ≥ 8% and < 8% in both follow‐up groups. Patients with initial higher HbA1c levels tended to have higher HbA1c levels at ophthalmology visits. Meanwhile, patients with higher DR scores had significantly higher rates of HbA1c levels ≥ 8% at the initial ophthalmology visit (Appendix 2).

### 3.3 Associations Between MR and Changes in HbA1c Levels and the Risk of DR Severity

Table 2 presents the crude descriptive analysis of subjective MR and objective HbA1c level changes between patients with low and high DR scores. Results were further stratified based on different ophthalmology clinic follow‐up durations, showing that MR was not significantly associated with DR severity in either group (shorter follow‐up group *p* = 0.720 and longer follow‐up group *p* = 0.861). In contrast, HbA1c levels at key time points, including at the time of DM diagnosis, at the first ophthalmology clinic visit, and changes in HbA1c levels over time, were found to be significantly and positively associated with DR severity.

**TABLE 2:** Descriptive results of subjective medication adherence and objective HbA1c level changes between low and high DR scores, stratified by different ophthalmology clinic follow‐up durations.

DR score	OPH‐OPD f/u duration < 5 years			OPH‐OPD f/u duration ≥ 5 years		
	0–1 points	2–3 points	*p*	0–1 points	2–3 points	*p*
Medication adherence			0.720			0.861
High	25 (48.08%)	30 (44.78%)		11 (52.38%)	19 (50.00%)	
Low and moderate	27 (51.92%)	37 (55.22%)		10 (47.62%)	19 (50.00%)	
HbA1c when DM diagnosed (%)			**< 0.001**			**0.006**
< 8%	34 (65.38%)	18 (26.87%)		17 (80.95%)	16 (42.11%)	
≥ 8%	18 (34.62%)	49 (73.13%)		4 (19.05%)	22 (57.89%)	
HbA1c at OPH OPD (%)			**0.006**			**0.011**
< 8%	40 (76.92%)	35 (52.24%)		20 (95.24%)	24 (63.16%)	
≥ 8%	12 (23.08%)	32 (47.76%)		1 (4.76%)	14 (36.84%)	
HbA1c level change^a^			**< 0.001**			**0.003**
Sustain low level (maintained at < 8%)	30 (57.69%)	13 (19.40%)		17 (80.95%)	12 (31.58%)	
Improved (decreased from ≥ 8% to < 8%)	10 (19.23%)	22 (32.84%)		3 (14.29%)	12 (31.58%)	
Deteriorated (increased from < 8% to ≥ 8%)	4 (7.69%)	5 (7.46%)		0 (0.00%)	4 (10.53%)	
Sustain high level (maintained at ≥ 8%)	8 (15.38%)	27 (40.30%)		1 (4.76%)	10 (26.32%)	

*Note*: Bold value indicates statistical significance *p < *0.05.

Abbreviations: DR, diabetic retinopathy; OPH‐OPD f/u duration, ophthalmology outpatient (OPH‐OPD) visit follow‐up duration.

^a^ Changes in HbA1c levels were determined based on laboratory data, using a cut‐off point of 8%. This change was assessed from the time of diabetes diagnosis to the first ophthalmologic visit and categorized into four groups: sustained low (remained < 8%), improved (decreased from ≥ 8% to < 8%), deteriorated (increased from < 8% to ≥ 8%), and sustained high (remained ≥ 8%).

Table 3 presents the univariable and multivariable logistic regression models showing the associations between MR and changes in HbA1c levels and the risk of DR severity. MR had an odds ratio of 0.92 in the multivariable model for DR severity risk. The only significant factor in univariate and multivariate analysis was age ≥ 65 years, which was identified as protective against higher DR severity (adjusted OR [aOR]: 0.43; 95% CI: 0.21–0.90; *p* = 0.025). A strong association was noted between changes in HbA1c levels and higher DR scores. Both improvements and deteriorations in HbA1c levels were associated with a higher likelihood of advanced DR.

**TABLE 3:** Univariate and multivariable models for examining the association between medical adherence, changes of HbA1c level, and DR severity.

	Model 1		Model 2		Model 3		Model 4	
	Univariable model		Multivariable model		Univariable model		Multivariable model	
	OR (95% CI)	*p*	AOR (95% CI)	*p*	OR (95% CI)	*p*	AOR (95% CI)	*p*
Adherence (ref: low and moderate)								
High	0.90 (0.50, 1.64)	0.728	0.92 (0.48, 1.75)	0.788				
HbA1c level change (ref = sustain low level)								
Lower (improve)					4.92 (2.20, 10.97)	< 0.001	5.42 (2.29, 12.80)	< 0.001
Higher (deteriorated) and sustain high level					6.65 (3.04, 14.57)	< 0.001	7.38 (3.02, 18.05)	< 0.001
Sex								
Male	Ref.		Ref.		Ref.		Ref.	
Female	1.05 (0.58, 1.90)	0.879	0.94 (0.48, 1.82)	0.843	1.05 (0.58, 1.90)	0.879	0.84 (0.41, 1.75)	0.643
Age categories								
< 65	Ref.		Ref.		Ref.		Ref.	
≥ 65	0.54 (0.30, 1.00)	0.049	0.43 (0.21, 0.90)	0.025	0.54 (0.30, 1.00)	0.049	0.62 (0.27, 1.40)	0.251
BMI	1.03 (0.97, 1.09)	0.393	1.01 (0.94, 1.08)	0.823	1.03 (0.97, 1.09)	0.393	1.02 (0.94, 1.09)	0.705
Education level								
Illiterate/elementary school	Ref.		Ref.		Ref.		Ref.	
Junior high school	0.80 (0.29, 2.21)	0.660	0.68 (0.23, 2.06)	0.498	0.80 (0.29, 2.21)	0.660	0.54 (0.16, 1.83)	0.323
High school/technical school	0.82 (0.37, 1.80)	0.615	0.61 (0.24, 1.51)	0.281	0.82 (0.37, 1.80)	0.615	0.59 (0.22, 1.59)	0.293
College or above	0.76 (0.29, 2.03)	0.587	0.57 (0.19, 1.74)	0.319	0.76 (0.29, 2.03)	0.587	0.50 (0.15, 1.68)	0.259
Marital status								
Single	Ref.		Ref.		Ref.		Ref.	
Married/cohabiting	0.62 (0.25, 1.56)	0.310	0.73 (0.27, 2.01)	0.544	0.62 (0.25, 1.56)	0.310	1.08 (0.37, 3.19)	0.887
Divorced/widowed	1.00 (0.33, 3.05)	1.000	1.20 (0.35, 4.11)	0.767	1.00 (0.33, 3.05)	1.000	1.60 (0.43, 5.93)	0.486
Diabetes duration (years)	1.03 (0.99, 1.07)	0.222	1.04 (0.99, 1.08)	0.141	1.03 (0.99, 1.07)	0.222	1.02 (0.97, 1.07)	0.409
Diabetes control methods								
No exercise	Ref.		Ref.		Ref.		Ref.	
Exercise	0.70 (0.38, 1.27)	0.234	0.87 (0.45, 1.70)	0.684	0.70 (0.38, 1.27)	0.234	0.93 (0.46, 1.92)	0.852
No diet	Ref.		Ref.		Ref.		Ref.	
Diet	1.60 (0.79, 3.26)	0.191	1.68 (0.78, 3.59)	0.185	1.60 (0.79, 3.26)	0.191	1.74 (0.75, 4.03)	0.193
Chronic disease comorbidity score group								
0 points	Ref.		Ref.		Ref.		Ref.	
1–2 points	0.81 (0.32, 2.05)	0.651	0.77 (0.28, 2.11)	0.609	0.81 (0.32, 2.05)	0.651	1.06 (0.35, 3.24)	0.916
3 points or above	0.76 (0.22, 2.60)	0.664	0.65 (0.16, 2.57)	0.536	0.76 (0.22, 2.60)	0.664	0.78 (0.17, 3.48)	0.744
OPH OPD f/u duration								
< 5 years	Ref.		Ref.		Ref.		Ref.	
≥ 5 years	1.40 (0.74, 2.68)	0.302	1.25 (0.61, 2.56)	0.546	1.40 (0.74, 2.68)	0.302	1.77 (0.80, 3.93)	0.162

*Note*: Model 1 and Model 2 were univariable and multivariable models examining the association between medical adherence and DR severity, while Model 3 and Model 4 assessed the association between HbA1c level changes and DR severity.

Abbreviations: AOR, adjusted odds ratio; BMI, body mass index; OPH‐OPD f/u duration, ophthalmology outpatient (OPH‐OPD) visit follow‐up duration; ref., reference group.

**FIGURE 1:**
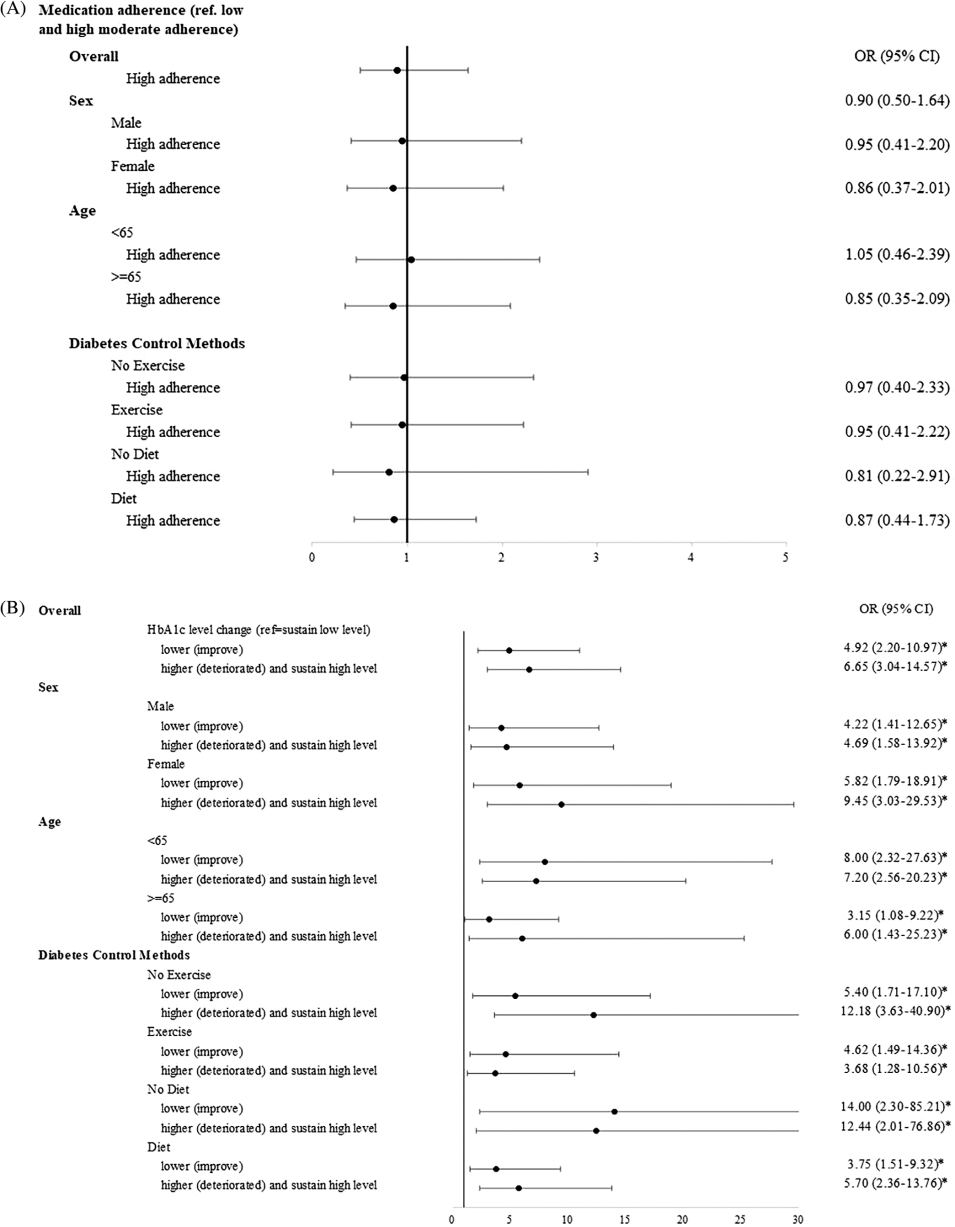
Forest plots for the association between medication adherence (A) and changes of HbA1c level (B) and the DR severity, stratifying by sex, age, and diabetes control methods.

Figure 1 presents forest plots illustrating the associations between MR (Figure 1A) and changes in HbA1c levels (Figure 1B) with DR severity, stratified by sex, age, and diabetes control methods (exercise, diet, or other methods). MR was not significantly associated with DR severity across different subgroups, including sex, age, and diabetes management strategies (exercise, diet, or other methods). In contrast, HbA1c changes were significantly associated with DR severity, with notable variations among subgroups. Females, younger individuals, non‐exercisers, and those without diet control had a higher risk of severe DR. These findings suggest that HbA1c fluctuations are critical in DR progression and that glycemic control's impact on DR severity may be influenced by demographic and lifestyle factors.

### 3.4 Risk Factors Associated With High HbA1c Levels

The present study further analyzed factors associated with HbA1c levels ≥ 8% at ophthalmology visits, using HbA1c < 8% as the reference. Both univariate and multivariate logistic regression models were applied, as detailed in Appendix 3. In univariate analysis, high medical adherence was significantly associated with a lower likelihood of patients having HbA1c levels ≥ 8% (crude OR: 0.48; 95% CI: 0.25–0.90; *p* = 0.023). However, this association did not remain significant in the full model (AOR: 0.56; 95% CI: 0.25–1.24; *p* = 0.152), indicating that the effect of adherence on HbA1c levels ≥ 8% may be confounded by other factors. Age ≥ 65 years was significantly associated with a lower likelihood of having an HbA1c level ≥ 8% in both models (crude OR: 0.24; 95% CI: 0.12–0.50; *p* < 0.001; AOR: 0.31; 95% CI: 0.12–0.84; *p* = 0.021). Higher chronic disease comorbidity scores were also significantly associated with a reduced risk of HbA1c levels ≥ 8%. In addition, HbA1c levels at diagnosis ≥ 8% remained a strong predictor of OPH‐OPD HbA1c levels ≥ 8% (AOR: 4.45; 95% CI: 1.75–11.29; *p* < 0.002) (Appendix 3).

## 4 Discussion

The present study, unlike previous landmark trials conducted in highly selected populations, evaluated these associations in a real‐world Asian cohort using routine clinical data, integrating glycemic indices with MR and sociodemographic factors. Additional variables, including diabetes duration, BMI, education, and social status, were also examined to provide a comprehensive understanding of risk determinants in everyday clinical practice.

Results of the present study showed strong associations between longitudinal HbA1c changes over the course of diabetes and STDR. Both improvements and deteriorations, along with sustained high HbA1c, were associated with greater DR severity compared to the sustained low group, with sustained high HbA1c having the most impact. High initial HbA1c had a stronger effect in females, patients under 65 years old, and those without exercise or diet control. Patients with low or moderate MR showed significantly higher HbA1c at the OPH visit, increasing their STDR risk. Age ≥ 65 years old was identified as a protective factor against higher DR scores.

High HbA1c is a well‐documented risk factor for DR [20]. The Wisconsin Epidemiology Study of Diabetic Retinopathy (WESDR) showed that higher levels of HbA1c increased the incidence and progression of DR [21]. Both the Diabetes Control and Complication Trial (DCCT) and the UKPDS found intensive glycemic control and reduction of HbA1c levels associated with decreased rates of development and progression of DR [22, 23]. This heightened risk is due to prolonged exposure to elevated blood sugar levels before diagnosis, which can cause long‐term retinal damage [24]. Unlike Type I diabetes, T2DM usually presents with non‐specific symptoms. Some patients were diagnosed with known diabetic complications, suggesting they may have had the disease for years before diagnosis. A meta‐analysis reported that the pooled prevalence of DR among patients with newly diagnosed T2DM was 13.1% [25].

Unlike other studies that investigated HbA1c at ophthalmic visits and the presence of DR, the present study focused on initial HbA1c at diabetes diagnosis and categories of DR according to DR status and the treatments received. High initial HbA1c may reflect a prolonged period of undiagnosed hyperglycemia, contributing to early retinal damage before formal diagnosis. It also implies the possibility of inappropriate living habits or poor compliance with health instruction. Unlike initial HbA1c, the above three possible factors are unmeasurable, emphasizing the importance of considering pre‐diagnosis glycemic exposure as a potential confounder when interpreting associations between initial HbA1c and DR progression, without diminishing its predictive value in clinical risk stratification. Recognizing high‐risk patients allows clinicians to consider closer early monitoring during the first years after diagnosis, while still incorporating long‐term glycemic trends into follow‐up decisions. This approach emphasizes that ophthalmology follow‐up should be individualized based on both initial and longitudinal glycemic control. In contrast, DM duration did not show a significant correlation with DR severity or progression in the present study, reflecting the clinical characteristics of T2DM and the often‐unreliable reported disease duration due to non‐specific symptom onset. The IMDIAB 25‐year follow‐up study also reported that HbA1c in the first year after diagnosis of T1DM is an independent risk factor for DR [26].

Although good glycemic control reduces DR risk, results of the present study showed that the improvement group had an increased likelihood of having STDR compared to the sustained low group, but less than the sustained high or deteriorated groups. Delayed diagnosis and high HbA1c may have lasting effects for years, and rapid glycemic control in patients with high initial HbA1c may trigger early worsening, characterized by macular edema and sudden vision deterioration, although this often improves over time [27].

The prognosis for DR in younger populations is often less favorable. An early onset of diabetes is an independent risk factor for developing PDR [28]. Although the mechanisms linking early‐onset diabetes to increased PDR risk are unclear, potential factors include variations in β‐cell function at different ages, increased concentrations of VEGF in younger individuals, and the complex psychosocial challenges faced by young adults [29, 30, 31]. Besides, younger individuals may skip medication or return visits due to work, family, or perceived wellbeing, resulting in poor glycemic control and faster DR progression. The Joint Asia Diabetes Evaluation (JADE) program also demonstrated that systemic metabolic control in young‐onset diabetes is worse than in late‐onset diabetes in Asia [32]. In contrast, older patients may have better adherence due to more stable routines and greater health literacy, contributing to more consistent diabetes management and a relatively better DR prognosis [33].

Although studies have shown that patients with comorbidities may have worse MR [34, 35], subgroup analysis in the present study showed that higher chronic disease comorbidity scores were significantly associated with a reduced risk of HbA1c levels ≥ 8%, consistent with our previous report ^40^, which found those with high complexity index (CIC) or diabetes complication severity index (DCSI) had better compliance [36]. This may be due to the broad coverage and extremely low co‐payment of our healthcare policy. Patients with comorbidities had greater insight into their disease, leading to consistent diabetes treatment. Although patients with higher comorbidity scores appeared to have slightly lower odds of developing severe DR, this association did not reach statistical significance, likely as a result of survivor bias, whereby patients with severe systemic comorbidities may have died before progressing to advanced stages of DR. Future studies with longitudinal follow‐up and survival analyses are warranted to further elucidate these potential mechanisms.

MR was associated with better HbA1c control but did not emerge as a statistically significant predictor of DR progression. In fact, results suggested that MR to prescribed medications alone may not be a determining factor in DR progression, possibly resulting from confounding variables such as baseline disease severity, genetic predisposition, or other unaccountable comorbidities [37]. In other words, adherence alone may not fully capture the complexity of DR pathogenesis, which involves multifactorial influences, including blood pressure, lipid levels, and other systemic factors beyond glycemic control [4, 38, 39, 40]. While adherence demonstrated some protective effects against high HbA1c levels, it did not consistently predict better DR outcomes in long‐term follow‐up, suggesting that adherence may be more effective in preventing early‐stage DR rather than halting progression in advanced cases.

In comparison to results of previous studies [33, 36], results of the present study did not observe significant gender differences in DR scores or MR. However, the odds ratio distribution for DR scores (2–3) compared to DR scores (0–1) in Figure 1 did highlight the risk of female on the effect of high HbA1c. The importance of exercise and diet control were also noticed in this distribution analysis.

This study has several limitations, including missing glycemic data, small sample size, patient selection bias, and treatment bias (tertiary referral center in southern Taiwan). MR was also assessed using the self‐reported MMAS‐8, which may be subject to recall and social desirability biases. Objective indicators, such as pharmacy refill records or electronic adherence data, were not available in this cohort because the dataset was limited to a single hospital. Moreover, patients could have refilled prescriptions at other healthcare facilities, resulting in incomplete adherence information. Nevertheless, the MMAS‐8 has been validated in similar populations and remains a widely used and practical instrument for evaluating MR in both clinical and community settings. Despite national health insurance covering 99.9% of the population of Taiwan with low co‐payment, the treatment criteria exist, and some patients do not adhere to recommended treatment due to various factors, including systemic and socio‐economic factors.

## 5 Conclusions

DR remains a global health challenge. Associations between initial blood glucose levels at diabetes diagnosis and subsequent changes during ophthalmology visits appear to influence DR severity and progression in the Asian population. High initial HbA1c may signal the need for closer early ophthalmic monitoring. Comprehensive management, including strict glycemic control and other systemic conditions, has the potential to significantly improve DR outcomes. Future research should focus on developing individualized, ethnicity‐specific strategies for more effective prevention and management of STDR.

## Funding

This work was supported by the Kaohsiung Medical University Hospital (A11264n, KMUH112‐2M35, and KMUH110‐0M47).

## Ethics Statement

The study protocol and instruments used for evaluations were approved by the Institutional Review Board (IRB) and Ethics Committee of Kaohsiung Medical University Hospital (IRB number: KMUHIRB‐E(II)‐20220116) in accordance with the Declaration of Helsinki. All included patients provided signed informed consent.

## Conflicts of Interest

The authors declare no conflicts of interest.

## Data Availability

The data that support the findings of this study are available in the Supporting Information.

## Endnotes

^1^
Use of the MMAS is protected by US copyright laws. Permission for use is required. A license to use the Morisky scales can be obtained through MMAR, LLC, dba adherence: Donald.morisky@moriskyscale.com; www.moriskyscale.com.

## Appendix 1 Demographic and Clinical Characteristics of the Study Population by Adherence Levels

**Table:**

	Total	High adherence	Moderate and low adherence	*p*
*N*	178	85 (47.75%)	93 (52.25%)	
Gender				0.453
Male	89 (50.00%)	45 (52.94%)	44 (47.31%)	
Female	89 (50.00%)	40 (47.06%)	49 (52.69%)	
Average age	61.67 ± 10.90	63.31 ± 11.10	60.18 ± 10.55	0.056
Age categories				0.200
< 65	101 (56.74%)	44 (51.76%)	57 (61.29%)	
≥ 65	77 (43.26%)	41 (48.24%)	36 (38.71%)	
BMI	26.14 ± 4.94	25.89 ± 4.75	26.37 ± 5.11	0.522
Education level				0.425
Illiterate/elementary school	38 (21.35%)	20 (23.53%)	18 (19.35%)	
Middle school	26 (14.61%)	9 (10.59%)	17 (18.28%)	
High school/vocational high school	84 (47.19%)	43 (50.59%)	41 (44.09%)	
Bachelor's or above	30 (16.85%)	13 (15.29%)	17 (18.28%)	
Marital status				0.496
Single	24 (13.48%)	9 (10.59%)	15 (16.13%)	
Married/cohabiting	121 (67.98%)	61 (71.76%)	60 (64.52%)	
Divorced/widowed	33 (18.54%)	15 (17.65%)	18 (19.35%)	
Diabetes duration (in years)	11.21 ± 7.85	11.97 ± 8.70	10.51 ± 6.94	0.223
Diabetes management (multiple choice)				
Exercise	88 (49.44%)	50 (58.82%)	38 (40.86%)	**0.017**
Diet	138 (77.53%)	70 (82.35%)	68 (73.12%)	0.140
Oral hypoglycemic drugs/insulin	177 (99.44%)	85 (100.00%)	92 (98.92%)	1.000
Average chronic disease comorbidity score	1.50 ± 0.87	1.56 ± 0.92	1.44 ± 0.83	0.345
Chronic disease comorbidity score				0.165
0	22 (12.36%)	9 (10.59%)	13 (13.98%)	
1–2	135 (75.84%)	62 (72.94%)	73 (78.49%)	
3	21 (11.80%)	14 (16.47%)	7 (7.53%)	
Chronic diseases (multiple choice)				
Hypertension	131 (73.60%)	64 (75.29%)	67 (72.04%)	0.623
Heart disease	36 (20.22%)	19 (22.35%)	17 (18.28%)	0.499
Stroke	13 (7.30%)	5 (5.88%)	8 (8.60%)	0.572
Kidney‐related diseases	55 (30.90%)	27 (31.76%)	28 (30.11%)	0.811
Liver‐related diseases	20 (11.24%)	11 (12.94%)	9 (9.68%)	0.491
Endocrine‐related diseases	12 (6.74%)	7 (8.24%)	5 (5.38%)	0.555
Others	158 (88.76%)	75 (88.24%)	83 (89.25%)	0.831
Blood glucose level when DM diagnosed				**0.043**
100–200	63 (35.39%)	38 (44.71%)	25 (26.88%)	
200–300	71 (39.89%)	30 (35.29%)	41 (44.09%)	
≥ 300	44 (24.72%)	17 (20.00%)	27 (29.03%)	
HbA1c when diabetes diagnosed (%)				**0.026**
< 8	85 (47.75%)	48 (56.47%)	37 (39.78%)	
≥ 8	93 (52.25%)	37 (43.53%)	56 (60.22%)	
HbA1c at OPH OPD (%)				**0.022**
< 8	119 (66.85%)	64 (75.29%)	55 (59.14%)	
≥ 8	59 (33.15%)	21 (24.71%)	38 (40.86%)	

*Note*: Bold value indicates statistical significance *p* < 0.05.

## Appendix 2 Descriptive Results of Patient Characteristics Among Different Group With HbA1c Level at OPH OPD Visits, Stratified by Different Ophthalmology Clinic Follow‐Up Durations

**Table:**

	OPH OPD f/u duration < 5 years				OPH OPD f/u duration ≥ 5 years			
	Overall	HbA1c at OPH OPD < 8%	HbA1c at OPH OPD ≥ 8%	*p*	Overall	HbA1c at OPH OPD < 8%	HbA1c at OPH OPD ≥ 8%	*p*
*N*	119	75 (63.03%)	44 (36.97%)		59	44 (74.58%)	15 (25.42%)	
Adherence				**0.042**				0.382
High	55 (46.22%)	40 (53.33%)	15 (34.09%)		30 (50.85%)	24 (54.55%)	6 (40.00%)	
Low and moderate	64 (53.78%)	35 (46.67%)	29 (65.91%)		29 (49.15%)	20 (45.45%)	9 (60.00%)	
Gender				0.497				0.325
Male	67 (56.30%)	44 (58.67%)	23 (52.27%)		22 (37.29%)	18 (40.91%)	4 (26.67%)	
Female	52 (43.70%)	31 (41.33%)	21 (47.73%)		37 (62.71%)	26 (59.09%)	11 (73.33%)	
Average age	60.01 ± 11.39				65.03 ± 9.02	67.07 ± 7.47	59.07 ± 10.73	**0.002**
Age categories				**0.009**				**0.001**
< 65	71 (59.66%)	38 (50.67%)	33 (75.00%)		30 (50.85%)	17 (38.64%)	13 (86.67%)	
≥ 65	48 (40.34%)	37 (49.33%)	11 (25.00%)		29 (49.15%)	27 (61.36%)	2 (13.33%)	
BMI	26.19 ± 4.70	26.34 ± 4.97	25.94 ± 4.22	0.642	26.04 ± 5.43	24.82 ± 3.96	29.59 ± 7.46	**0.030**
Education level				0.573				0.443
Illiterate/elementary school	19 (15.97%)	14 (18.67%)	5 (11.36%)		19 (32.20%)	16 (36.36%)	3 (20.00%)	
Junior high school	17 (14.29%)	12 (16.00%)	5 (11.36%)		9 (15.25%)	5 (11.36%)	4 (26.67%)	
High school/technical school	60 (50.42%)	36 (48.00%)	24 (54.55%)		24 (40.68%)	18 (40.91%)	6 (40.00%)	
College or above	23 (19.33%)	13 (17.33%)	10 (22.73%)		7 (11.86%)	5 (11.36%)	2 (13.33%)	
Marital status				0.070				0.871
Single	18 (15.13%)	7 (9.33%)	11 (25.00%)		6 (10.17%)	4 (9.09%)	2 (13.33%)	
Married/cohabiting	79 (66.39%)	53 (70.67%)	26 (59.09%)		42 (71.19%)	32 (72.73%)	10 (66.67%)	
Divorced/widowed	22 (18.49%)	15 (20.00%)	7 (15.91%)		11 (18.64%)	8 (18.18%)	3 (20.00%)	
Diabetes duration (years)	9.58 ± 7.10	9.61 ± 6.92	9.52 ± 7.46	0.947	14.49 ± 8.30	14.61 ± 8.37	14.13 ± 8.38	0.849
Diabetes control methods (multiple choice)								
Exercise	60 (50.42%)	38 (50.67%)	22 (50.00%)	1.000	28 (47.46%)	25 (56.82%)	3 (20.00%)	**0.018**
Diet	91 (76.47%)	54 (72.00%)	37 (84.09%)	0.180	47 (79.66%)	36 (81.82%)	11 (73.33%)	0.479
Oral hypoglycemic drugs/insulin	118 (99.16%)	75 (100.00%)	43 (97.73%)	0.370	59 (100.00%)	44 (100.00%)	15 (100.00%)	—
Average chronic disease comorbidity score	1.46 ± 0.90	1.57 ± 0.81	1.27 ± 1.02	0.079	1.58 ± 0.81	1.55 ± 0.87	1.67 ± 0.62	0.562
Chronic disease comorbidity score group				**0.003**				0.394
0 points	18 (15.13%)	5 (6.67%)	13 (29.55%)		4 (6.78%)	4 (9.09%)	0 (0.00%)	
1–2 points	86 (72.27%)	60 (80.00%)	26 (59.09%)		49 (83.05%)	35 (79.55%)	14 (93.33%)	
3 points or above	15 (12.61%)	10 (13.33%)	5 (11.36%)		6 (10.17%)	5 (11.36%)	1 (6.67%)	
History of chronic diseases (multiple choice)								
Hypertension	83 (69.75%)	60 (80.00%)	23 (52.27%)	**0.002**	48 (81.36%)	35 (79.55%)	13 (86.67%)	0.712
Heart disease	23 (19.33%)	17 (22.67%)	6 (13.64%)	0.336	13 (22.03%)	10 (22.73%)	3 (20.00%)	1.000
Stroke	8 (6.72%)	4 (5.33%)	4 (9.09%)	0.466	5 (8.47%)	4 (9.09%)	1 (6.67%)	1.000
Kidney‐related diseases	37 (31.09%)	22 (29.33%)	15 (34.09%)	0.682	18 (30.51%)	11 (25.00%)	7 (46.67%)	0.192
Liver‐related diseases	13 (10.92%)	8 (10.67%)	5 (11.36%)	1.000	7 (11.86%)	7 (15.91%)	0 (0.00%)	0.174
Endocrine‐related diseases	10 (8.40%)	7 (9.33%)	3 (6.82%)	0.743	2 (3.39%)	1 (2.27%)	1 (6.67%)	0.447
Others	104 (87.39%)	64 (85.33%)	40 (90.91%)	0.568	54 (91.53%)	40 (90.91%)	14 (93.33%)	0.771
Blood glucose level when DM diagnosed				**< 0.001**				0.222
100–200	41 (34.45%)	36 (48.00%)	5 (11.36%)		22 (37.29%)	19 (43.18%)	3 (20.00%)	
200–300	49 (41.18%)	23 (30.67%)	26 (59.09%)		22 (37.29%)	14 (31.82%)	8 (53.33%)	
≥ 300	29 (24.37%)	16 (21.33%)	13 (29.55%)		15 (25.42%)	11 (25.00%)	4 (26.67%)	
HbA1c when DM diagnosed				**< 0.001**				**0.015**
< 8	52 (43.70%)	43 (57.33%)	9 (20.45%)		33 (55.93%)	29 (65.91%)	4 (26.67%)	
≥ 8	67 (56.30%)	32 (42.67%)	35 (79.55%)		26 (44.07%)	15 (34.09%)	11 (73.33%)	
DR score				**0.006**				**0.011**
0–1	75 (63.03%)	40 (53.33%)	12 (27.27%)		44 (74.58%)	20 (45.45%)	1 (6.67%)	
2–3	44 (36.97%)	35 (46.67%)	32 (72.73%)		15 (25.42%)	24 (54.55%)	14 (93.33%)	

*Note*: Bold value indicates statistical significance *p* < 0.05.

## Appendix 3 Univariate and Multivariable Logistic Regression Models Assessing the Association Between Medication Adherence and HbA1c Levels (≥ 8% vs. < 8%), as Well as Related Risk Factors

**Table:**

	HbA1c at OPH OPD ≥ 8% (ref = < 8%)			
	Univariate model		Multivariable model	
	Overall		Overall	
	OR (95% CI)	*p*	OR (95% CI)	*p*
Adherence (ref = low and moderate)				
High	0.48 (0.25, 0.90)	0.023	0.56 (0.25, 1.24)	0.152
Sex (ref = male)				
Female	1.29 (0.69, 2.41)	0.426	1.56 (0.66, 3.68)	0.310
Age categories (ref = < 65)				
≥ 65	0.24 (0.12, 0.50)	< 0.001	0.31 (0.12, 0.84)	0.021
BMI	1.05 (0.98, 1.11)	0.168	1.03 (0.96, 1.12)	0.394
Education level (ref = illiterate/elementary school)				
Junior high school	1.99 (0.65, 6.10)	0.231	2.59 (0.63, 10.68)	0.188
High school/technical school	2.08 (0.85, 5.12)	0.109	1.64 (0.50, 5.42)	0.417
College or above	2.50 (0.86, 7.28)	0.093	1.43 (0.34, 6.00)	0.625
Marital status (ref = single)				
Married/cohabiting	0.36 (0.15, 0.88)	0.024	0.97 (0.31, 2.99)	0.950
Divorced/widowed	0.37 (0.12, 1.10)	0.073	0.82 (0.21, 3.26)	0.780
Diabetes duration (years)	0.99 (0.95, 1.03)	0.538	1.02 (0.96, 1.08)	0.462
Diabetes control methods (ref = no)				
Exercise	0.65 (0.35, 1.23)	0.186	1.30 (0.56, 3.01)	0.537
Diet	1.41 (0.65, 3.06)	0.390	1.67 (0.62, 4.51)	0.310
Chronic disease comorbidity score group (ref = 0)				
1–2 points	0.29 (0.12, 0.74)	0.009	0.31 (0.10, 0.97)	0.044
3 points or above	0.28 (0.08, 0.99)	0.048	0.26 (0.05, 1.48)	0.129
Blood glucose level when DM diagnosed (ref = 100–200)				
200–300	6.32 (2.63, 15.16)	< 0.001	2.42 (0.84, 6.96)	0.102
≥ 300	4.33 (1.66, 11.28)	0.003	0.98 (0.29, 3.39)	0.978
HbA1c level when DM diagnosed (ref = < 8)				
≥ 8	5.42 (2.65, 11.10)	< 0.001	4.45 (1.75, 11.29)	0.002
DR score (ref = 0–1)				
2–3	3.60 (1.76, 7.34)	< 0.001	2.32 (0.93, 5.76)	0.071
OPH OPD f/u duration (ref = < 5 years)				
≥ 5 years	0.58 (0.29, 1.16)	0.126	0.59 (0.23, 1.50)	0.267
